# Impact of acute-on-chronic liver failure and decompensated liver cirrhosis on psychosocial burden and quality of life of patients and their close relatives

**DOI:** 10.1186/s12955-019-1268-9

**Published:** 2020-01-13

**Authors:** Michael Nagel, Christian Labenz, Marcus A. Wörns, J. U. Marquardt, Peter R. Galle, Jörn M. Schattenberg, Marc Nguyen-Tat

**Affiliations:** 1 First Department of Medicine, University Medical Center Mainz of the Johannes Gutenberg-University Mainz, Langenbeckstrasse 1, 55131 Mainz, Germany; 2 Cirrhosis Center Mainz (CCM), University Medical Center of the Johannes Gutenberg-University Mainz, Mainz, Germany; 3 Lichtenberg Research Group for Molecular Hepatocarcinogenesis, University Medical Center of the Johannes Gutenberg-University Mainz, Mainz, Germany; 4 Metabolic Liver Research Program, University Medical Center of the Johannes Gutenberg-University Mainz, Mainz, Germany; 5 Medical Center Osnabrück, Department of Internal Medicine II, Am Finkenhügel 1, 49076 Osnabrück, Germany

**Keywords:** Liver cirrhosis, Acute – on – chronic liver failure, Psychosocial burden of relatives, Quality of life

## Abstract

**Background:**

Patients with liver cirrhosis often suffer from complications such as ascites, gastrointestinal bleeding, and infections, resulting in impaired quality of life. Frequently, the close relatives of patients also suffer from a lower quality of life in chronic diseases. In recent years, acute-to-chronic liver failure has been defined as a separate entity with high mortality. Often several organs are affected which makes intensive care therapy necessary. Little is known about the influence of acute-on-chronic-liver failure (ACLF) on the quality of life of patients and the psychosocial burden on close relatives.

**Aim:**

The purpose of this prospective study is to investigate the influence of decompensated liver cirrhosis and the onset of ACLF of the patient’s’ quality of life and the psychosocial burden of close relatives.

**Method:**

In this non – randomized prospective cohort study a total of 63 patients with acute decompensation of liver cirrhosis and hospital admission were enrolled in the study. To assess the quality of life of patients, the disease specific CLDQ questionnaire was assessed. In addition. Quality of life and psychosocial burden of first degree relatives was measured using the generic SF-36 questionnaire as well as the Zarit Burden Score.

**Results:**

21 of the 63 patients suffered from ACLF. Patients with ACLF showed a lower quality of life in terms of worries compared to patients with only decompensated liver cirrhosis (3,57 ± 1,17 vs. 4,48 ± 1,27; *p* value: 0,008) and increased systemic symptoms (3,29 ± 1,19 vs. 4,48 ± 1,58; *p* value: 0,004). The univariate analysis confirmed the link between the existence of an ACLF and the concerns of patients. (*p* value: 0,001). The organ failure score was significantly associated with overall CLDQ scores, especially with worries and systemic symptoms of patients. Interestingly the psychosocial burden and quality of life of close relative correlates with patient’s quality of life and was influenced by the onset of an acute-on-chronic liver failure.

**Conclusion:**

Patients with decompensated liver cirrhosis suffer from impaired quality of life. In particular, patients with ACLF have a significantly reduced quality of life. The extent of the psychosocial burden on close relative correlates with poor quality of life in patients with decompensated liver disease and is influenced by the existence of ACLF.

## Introduction

Chronic liver disease is a relevant cause of morbidity and mortality worldwide. Every year, more than one million patients die worldwide as a result of liver cirrhosis [1]. In particular the acute-on-chronic liver failure is associated with a bad outcome. Due to the high short-term mortality, acute-on-chronic liver failure is not only a therapeutic challenge but also a burden for patients and their relatives. In recent years, studies have shown that quality of life in patients with chronic liver disease and especially liver cirrhosis is significantly impaired [2]. The degree of impairment of quality of life measured by CLDQ is associated with survival, especially when ascites occurred [3]. However, data from studies on quality of life in patients with acute-on-chronic liver failure is lacking. In addition, little is known about the impact on quality of life of patient’s relatives and caregivers in the setting of acute decompensation of liver cirrhosis. Evidence from studies of patients and caregivers in other chronic diseases like cancer points towards a significant impairment in quality of life in caregivers [4]. Psychosocial stress is increased in relatives of patients with liver cirrhosis [5]. Some complications of liver cirrhosis such as the occurrence of hepatic encephalopathy seem to cause particularly intense stress for caregiver [6]. In addition to optimal medical care, patients also benefit from intensive medical education and psychological therapy for their relatives [7]. Data on quality of life and psychosocial stress of patients with ACLF and their caregivers is scarce. Aim of this study was to assess the impact of acute decompensation of liver cirrhosis and ACLF on quality of life of patients and their closest relatives and caregivers as well as the impact on psychosocial health in caregivers.

## Material and methods

### Patient population

A total of 102 patients with decompensated liver cirrhosis who were hospitalized between May 2017 and May 2018 at the Cirrhosis Centre Mainz (CCM) of the University Medical Centre of the Johannes Gutenberg-University in Mainz, Germany were screened and 63 were enrolled. A planned admission was an exclusion criterion. In addition, patients without relatives were excluded too. Patients who could not give their consent due to the severity of the disease were also excluded. Patients with hepatocellular carcinoma or severe chronic diseases of other organ systems were also excluded. 21 patients fulfilled the criteria for the diagnosis of ACLF during the course of inpatient treatment. In all patients, diagnosis of liver cirrhosis had already been diagnosed by ultrasound, radiologically or by biopsy before presentation. At presentation, all patients received a standardized medical history, ultrasound and a laboratory examination. In addition to general epidemiological data such as age and gender, etiologies were classified as follows. Alcoholic cirrhosis due to chronic alcohol consumption based on biopsy and medical history, non-alcoholic steatohepatitis due to histological findings and presence of cardiovascular risk factors, viral cirrhosis with chronic HBV, HDV and HCV infections based on laboratory findings. Cholestatic/autoimmune liver cirrhosis includes autoimmune hepatitis, primary sclerosing cholangitis, primary biliary cholangitis and secondary sclerosing cholangitis diagnosed by medical histology, radiological and laboratory results. Metabolic/hereditary liver cirrhosis with hemochromatosis, Morbus Wilson and alpha1-antitrypsin deficiency were diagnosed by histological findings and laboratory changes as well as vascular liver cirrhosis with Budd-Chiari syndrome or portal hypertension with portal vein thrombosis by histology findings. If no cause could be found, liver cirrhosis was classified as cryptogenic.

### Diagnostic criteria of acute on chronic liver failure

From first day at admission, daily calculation of ACLF degree, ACLF score and organ failure were performed. In addition, the acute decompensation score was calculated daily in patients with decompensated cirrhosis without fulfilling the ACLF criteria. To determine ACLF stage, the specifications of the CLIF consortium were used: serum bilirubin ≥12 mg/dL; kidney failure: serum creatinine ≥2 mg/dL or use of hemodialysis; cerebral failure: grade III-IV hepatic encephalopathy (West-Haven classification); coagulation failure: international normalized ratio (INR) ≥2.5 and/or platelets < 20.000/μL; circulatory failure: use of vasopressors to treat severe arterial hypotension. Respiratory failure: PaO2/FiO2 ≤ 200 or SpO2/ FiO2 ≤ 214. Stage 1 ACLF (ACLF 1) is defined by the presence of renal failure alone or of any other type of single organ failure if associated with renal dysfunction (serum creatinine between 1.5 and 1.9 mg/dL) and/or cerebral dysfunction (Grade I or Grade II hepatic encephalopathy). Stage II ACLF and Stage III ACLF define the presence of 2 and 3 to 6 organ failures, respectively. The maximum OF and ACLF score during treatment were used for classification into the comparison groups and for further calculation.

### Assessment of quality of life of patients and caregivers

To assess quality of life we used the validated German version of the Chronic Liver Disease Questionnaire (CLDQ). The questionnaire contains 29 items which can be grouped into the liver-disease specific domains activity, fatigue, worries, abdominal symptoms, and systemic symptoms. Each category can be assessed separately between groups. Higher results indicate better quality of life [8]. Closest relatives and caregivers of the patients were asked about their quality of life and psychosocial health. The Zarit Burden Score was used to determine psychosocial stress [5]. The quality of life of relatives and caregivers was assessed using the SF-36 questionnaire [9].

### Ethics

The study was conducted according to the ethical guidelines of the 1975 Declaration of Helsinki (6th revision, 2008). The study protocol was approved by the ethics committee of the Landesärztekammer Rhineland-Palatine (Nr. 837.232.17 [11066]). Written informed consent was obtained from every participant.

### Statistical analysis

The statistical analyses were performed with SPSS Version 23. Continuous variables are presented as means with standard deviation. Categorial variables are presented as frequencies and percentages. Categorial variables were compared using Chi-Quadrat test or fisher’s exact test, and continuous variables were compared using student’s T–Test or Mann-Whitney U-Test. *P* Value below 0.05 was considered to be statistically significant.

## Results

### Patient baseline characteristics

A total of 63 patients were included. 21 patients had ACLF and 42 patients had decompensated liver cirrhosis without meeting the ACLF criteria. Alcohol liver cirrhosis was the most common underlying cause of liver cirrhosis (21% for ACLF vs. 41% for decompensated liver cirrhosis) followed by NASH associated liver cirrhosis (10% for ACLF vs. 16% for decompensated liver cirrhosis). There was no significant difference between ACLF and decompensated liver cirrhosis with respect to the etiology of liver cirrhosis (*P* value: 0,7). Patients with ACLF had significantly higher MELD (15 + 6 vs. 22 + 7) and organ failure scores (7 ± 1 vs. 10 ± 2) than patients with decompensated liver cirrhosis (*P* value: < 0,001) (Table 1).

**Table 1:** Patient characteristics. The most common etiology was alcoholic liver cirrhosis (41% vs. 22%) in both groups followed by NASH (16% vs. 10%; *p* value 0,7). Impairment of liver function was higher in patients with ACLF measured by MELD Score (15 ± 6 vs. 22 ± 7; *p* value < 0,001) and Organ Failure Score (7 ± 1 vs. 10 ± 2; *p* value < 0,001)

Parameter	Decompensated liver cirrhosis (*N* = 42)	Acute-on-chronic liver failure (*N* = 21)	*P* Value
Male Gender (N; %)	25 (40%)	13 (21%)	0,9
Age (years) (MEAN; SD)	58 ± 14	58 ± 10	0,9
Etiology			0,7
*alcoholic* (N; %)	26 (41%)	14 (22%)	
*cryptogen/NASH* (N; %)	10 (16%)	6 (10%)	
*Viral* (N; %)	3 (5%)	0 (0%)	
*cholestatic/ autoimmune* (N; %)	2 (3%)	1 (2%)	
*metabolic/hereditary* (N; %)	1 (2%)	0 (0%)	
MELD (MEAN; SD)	15 ± 6	22 ± 7	< 0,001
Organ Failure Score (MEAN; SD)	7 ± 1	10 ± 2	< 0,001

### CLDQ is severely impaired in patients with ACLF

Quality of life of patients with ACLF and decompensated cirrhosis was assessed by CLDQ. (Table 2 and 3). In patients with acute-on-chronic liver failure quality of life was significantly impaired compared to patients with decompensated liver cirrhosis (4,38 ± 1,14 vs. 3,67 ± 0,91; *p* value: 0,02). In particular burden from systematic symptoms (4,5 ± 1,6 vs. 3,3 ± 1,3; *p* value: 0,004) and worries (4,5 ± 1,3 vs. 3,6 ± 1,2; *p* value 0,008) was higher in patients with ACLF.

**Table 2:** Shows the quality of life of patients with aspects of fatigue, emotional function, worries, abdominal symptoms, activity and systemic symptoms assessed by CQLD. Patients with ACLF showed a significantly reduced quality of life in terms of worry (4,48 ± 1,3 vs. 3,57 ± 1,2) and systemic symptoms (4,48 ± 1,6 vs. 3,29 ± 1,2)

Parameter	Decompensated liver cirrhosis (*N* = 42)	Acute-on-chronic liver failure (*N* = 21)	*P* Value
Fatigue (MEAN; SD)	3,98 ± 1,12	3,76 ± 1,09	0,5
Emotional Function (MEAN; SD)	4,24 ± 1,14	3,67 ± 1,07	0,06
Worries (MEAN; SD)	4,48 ± 1,27	3,57 ± 1,17	0,008
Abdominal symptoms (MEAN; SD)	4,43 ± 1,4	3,9 ± 1,22	0,2
Activity (MEAN; SD)	4,43 ± 1,31	4,1 ± 1,55	0,4
Systemic symptoms (MEAN; SD)	4,48 ± 1,58	3,29 ± 1,19	0,004
Total Quality of Life (MEAN; SD)	4,33 ± 1,14	3,67 ± 0,91	0,02

**Table 3:** Shows the univariate analysis in patients with ACLF. In addition to clinical factors such as CHILD status (*p* value: 0,03), hepatorenal syndrome as a cause of decompensation (*p* value: 0,007), duration of hospitalization (*p* value: 0,03) and intensive care therapy (*p* value: 0,04), the analysis also showed an influence on patients’ quality of life. The partial aspect of patient’s worries shows a significant association with the presence of ACLF (*p* value: 0,04)

Univariant analysis - ACLF				
Parameter	OR	95% - CI		*P* Value
CHILD Status	0,44	0,211	0,931	0,03
hepatorenal syndrome	20,5	2314	181,596	0,007
worries of patient	1,79	1133	2817	0,01
Duration of hospitalization	0,94	0,886	0,994	0,03
ICU therapy	0,16	0,028	0,911	0,04

### Patient’s quality of life is associated with degree of organ failure

The maximum Organ Failure Score during the inpatient stay was calculated for the patients (Table 4). The OF score correlates with patients’ quality of life. There is a significant correlation between quality of life issues such as fatigue (*r* = − 0,294, *p* value 0,002), emotional function (*r* = − 0,270, *p* value: 0,003), worry (*r* = − 0,420, *p* value: < 0,0001), activity (*r* = − 0,256, *p* value: 0,004) and systematic symptoms (*r* = − 0,358; *p* value: 0,003) with the OF score in ACLF patients.

**Table 4:** Shows the correlation of the individual aspects of the patients’ quality of life with the Organ Failure score. All partial aspects except abdominal symptoms like fatigue (*r* = −0,294; *p* value: 0,02), emotional function (*r* = − 0,27; *p* value: 0,03), worries (*r* = − 0,42; *p* value: < 0,001), activity (*r* = − 0,256; *p* value: 0,042) and systemic symptoms (− 0,358; *p* value: 0,003) showed a significant correlation with organ failure score

Correlation with Organ Failure Score		
Parameter	Correlation	*P* Value
Fatigue	- 0,294	0,02
Emotional function	- 0,270	0,03
Worries	- 0,420	< 0,001
Abdominal symptoms	- 0,152	0,2
Activity	- 0,256	0,04
Systemic symptoms	- 0,358	0,003

### The psychosocial burden and quality of life of close relative is independent of the presence of an ACLF

Psychosocial stress and quality of life of relatives were also assessed and compared between patients with decompensated cirrhosis and ACLF (Table 5). Interestingly, no significant differences were found in the investigated patient population. There was no significant difference in both psychosocial stress and quality of life in caregivers measured by physical, psychical, mental, and social strength. However, the univariate analysis showed a correlation with the age (OR: − 0,35; *p* value: 0,049; 95%-CI: − 0,69- − 0,001) of the close relatives, sodium of patients (OR: − 0,87; *p* value: 0,04; 95%-CI: − 1,7 - − 0,03) and the occurrence of hepatic encephalopathy (OR: 10,6; *p* value: 0,02; 95%-CI: 2,05-19,13). The presence of ACLF or impaired liver function had no effect on the psychosocial burden of the close relatives. In contrast, there was a clearly significant association with patient’s quality of life with psychosocial stress of relatives. Fatigue (OR: − 6,5; *p* value: 0,0004; 95%-CI: − 9,7- --3,2), emotional function (OR: − 7,5; *p* value: < 0,0001; 95%-CI: − 10,2 - -4,6), worries (OR: − 7,2; *p* value: < 0,0001; 95%-CI: − 9,6 - -4,8), abdominal symptoms (OR: − 5,1; *p* value: 0,0002; 95%-CI: − 7,7 - -2,6) as well as activity (OR: − 5,7; *p* value: < 0,0001; 95%-CI: − 8,1 - -3,2) and systematic symptoms (OR: − 0,3; *p* value: 0,002; 95%-CI: − 0,5 - − 0,13) showed a significant association with psychosocial burden in caregivers and close relatives (Table 6).

**Table 5:** Showed the quality of life of the relatives and the psychosocial burden. There is no difference between the quality of life and psychosocial burden of relatives of patients with ACLF or decompensated cirrhosis

Parameter	Decompensated liver cirrhosis (*N* = 42)	Acute-on-chronic liver failure (*N* = 21)	*P* Value
Psychosocial burden of relatives (MEAN; SD)	19,9 ± 11,5	25,9 ± 14,1	0,18
physical strength of relatives (MEAN; SD)	77,5 ± 18,9	69,5 ± 21,9	0,27
mental strength of relatives (MEAN; SD)	67,2 ± 12,9	61,6 ± 11,9	0,19
social strength of relatives (MEAN; SD)	68,4 ± 15,2	65,4 ± 19,3	0,62
Environmental strength of relatives (MEAN; SD)	77,5 ± 14,8	70,6 ± 16,4	0,21

**Table 6:** Shows the univariate analysis of psychosocial burden of relatives. Interestingly, there is a significant influence of the patient’s age (*p* value: 0,05), sodium (*p* value: 0,04) and hepatic encephalopathy (*p* value: 0,02) on the psychosocial burden of the relative. In addition, all aspects of impaired quality of life of patients such as fatigue (*p* value: 0,0004), emotional function (*p* value: < 0,00001), worries (*p* value: < 0,00001), abdominal symptoms (*p* value: 0,0002), patient activity (*p* value: 0,00005) and systemic symptoms (*p* value: 0,002) are associated with increased psychosocial stress in relatives

Univariate analysis – psychosocial burden of relatives				
Parameter	OR	95% - CI		*P* Value
Age	−0,35	-0,690	-0,001	0,05
Sodium	-0,87	− 1704	−0,030	0,04
hepatic encephalopathy	10,59	20,544	19,125	0,02
fatigue of patient	−6,52	− 9869	− 3179	0,0004
Emotional function of patient	−7,48	−10,333	− 4622	< 0,0001
worries of patient	− 7238	− 9618	− 4847	< 0,0001
Abdominal symptoms of patients	−5,14	− 7669	− 2611	0,0002
Activity of patient	−5,66	− 8126	− 3188	< 0,0001
Systemic symptoms of patient	−0,32	− 0,510	−0,129	0,002

## Discussion

In this prospective cohort study, the influence of acute on chronic liver failure on the quality of life of patients and their relatives was investigated for the first time. Patients with ACLF showed a significantly lower quality of life compared to the control group. In particular, the sub-domains of worries and systematic symptoms were decisive in patients with ACLF. That quality of life is an important factor in patients with chronic liver diseases, which has been shown in several studies [2]. Patients with decompensated liver cirrhosis show significantly worse values for quality of life. In our collective the patients with ACLF showed a higher MELD value and thus a more impaired liver function than the comparison group. Interestingly, the Organ Failure Score also correlates with quality of life. All subdomains of quality of life except abdominal symptoms show a significant correlation with the OF score. The OF score covers renal, hepatic, respiratory, cognitive, cardio-circulatory and coagulation functions. By definition, this function is more often limited in patients with ACLF than in patients with decompensated liver cirrhosis. As a systemic disease, ACLF has a much greater impact on the quality of life of patients. This systematic disease is mainly responsible for the significant limitation of the subdomain of systematic symptoms. Often younger patients with previously unknown liver cirrhosis suffer from ACLF in the sense of an initial diagnosis [10]. These patients have often not yet been able to deal adequately with their disease. In addition, many of these patients lack basic information about their disease. This is a possible cause of the reduced sub-domain of concern in these patients. These results have provided initial evidence that patients with ACLF suffer from a significantly reduced quality of life. In recent years awareness of impaired quality of life in patients with chronic liver disease, in particular liver cirrhosis has increased. The extent of the limitation of quality of life is directly related to the severity of the underlying liver disease [11, 12]. Especially in patients with refractory ascites degree of quality of life impairment has been shown to provide valuable information on 1-year mortality [3].

Besides the reduced quality of life of the patients, we were one of the first to show that the relatives of patients with ACLF suffer from a reduced quality of life. Both the psychological and physical strength of the relatives was reduced in our study. In addition, we were able to show that the psychosocial burden on the relatives of patients with ACLF is also significantly elevated. This reduced quality of life has an impact of the clinical course of the patients. Frequently, relatives are the first who register initially subtle changes in patients, for example in hepatic encephalopathy, and initiate first steps of therapy [13]. Studies on psychosocial stress of caregivers in chronic diseases such as dementia or ALS have shown that the degree of psychosocial stress even correlates with mortality in relatives and caregivers [14]. Investigations of psychosocial burden on relatives of patients with liver cirrhosis are rare. Bajaj et al. were able to show significant amounts of stress in relatives of patients with cirrhosis of the liver [6], especially if an episode of hepatic encephalopathy had preceded. In our study even higher levels of psychosocial stress were measured. On the one hand, this could be due to the fact that in our study all patients were recruited during inpatient treatment for acute decompensation of liver cirrhosis and were severely ill, with a significance percentage of patients with ACLF. Similar tendencies could be observed in all areas of quality of life of relatives. Our study provides early insights into the psychosocial burden and on daily quality of life in caregivers and relatives of patients with liver cirrhosis. So far to our knowledge no evidence-based interventions to support close relatives of patients with liver cirrhosis have been developed, however our data prove the need to not only care for these patients but also consider the burden placed on close relatives.

And we were also able to show in our study that the quality of life of the patient is closely correlated with the psychosocial stress of the relatives. All sub-domains of the quality of life showed a significant influence on the psychosocial load. Especially the limited activity, the worries and the emotional function correlate with the psychosocial stress. It is known that especially cognitive defects and reduction of vigilance, as in hepatic encephalopathy, have an influence on the psychosocial burden on relatives [15]. In our study the presence of hepatic encephalopathy was confirmed as an influencing factor to increase the burden on relatives. Hyponatremia is also a factor which was significantly associated with reduction of quality of life. Hyponatremia is often associated with advanced liver insufficiency and reduced vigilance. The combination of frequent and increased physical symptoms as well as the significantly reduced quality of life in patients with ACLF have a particularly strong influence on the quality of life and psychosocial stress of relatives.

It must be said that this is a small cohort of patients. Although there is a clear trend towards a restricted quality of life among relatives, but without significance. The small number of cases certainly plays a role here. In addition, patients with ACLF show significantly poorer liver function from the outset, so that this study should be repeated on a larger collective with matching by liver function. Nevertheless, we were able to show first indications of quality of life and psychosocial stress in patients with ACLF and their relatives. Influencing the quality of life and psychosocial stress should be an elementary part of the therapy of this patient. In order to improve the prognosis of these patients, greater emphasis should be placed on disease management and above all on training relatives to prevent elementary complications.

## Conclusion

Patients with acute-on-chronic liver failure suffer from a severe impairment in quality of life. This impairment is associated with the severity of organ failure measured by the Organ Failure Score. Close relatives and caregivers of patients with decompensated liver cirrhosis and ACLF suffer from psychosocial stress and impaired quality of life which is associated with severeness of impairment in patients with liver cirrhosis. Future research should focus not only on functional and psychosocial impairment in patients with liver cirrhosis but also on the development of evidence-based interventions to support close relatives of patients with liver cirrhosis.

## Data Availability

The data supporting the conclusion of this article are includes within the article. Any queries regarding these data may be directed to the corresponding author.

## Abbreviations

ACLF: Acute on chronic liver failure
CLDQ: Chronic liver disease questionnaire
CLIF Consortium: Foundation for the study of chronic liver failure
INR: International normalized ratio
OF Score: Organ failure score
SF-36: Short form health survey
